# Mechanistic investigation of Midkine-Notch2 signaling in suppressing pulmonary artery smooth muscle cell apoptosis and promoting vascular remodeling in hypoxic pulmonary hypertension

**DOI:** 10.3389/fphar.2026.1788582

**Published:** 2026-07-22

**Authors:** Houfan Zhu, Binglong Li, Xunkai Wang, Lin Huang, Jin Peng, Jiangpeng Wu, Yong Liao, Shengchao Wei, Shijie Zhong, Guiyun Jin, Tang Deng

**Affiliations:** 1 Key Laboratory of Emergency and Trauma of Ministry of Education, Department of Interventional Radiology and Vascular Surgery, The First Affiliated Hospital of Hainan Medical University, Hainan Medical University, Haikou, China; 2 Division of Vascular Surgery, The First Affiliated Hospital, Sun Yat-sen University, Guangzhou, China; 3 Department of Radiology, Hainan Modern Women and Children’s Hospital, Haikou, China

**Keywords:** apoptosis, hypoxic pulmonary hypertension (HPH), Midkine, Notch2 signaling pathway, pulmonary vascular remodeling

## Abstract

**Introduction:**

Hypoxic pulmonary hypertension (HPH), a severe complication of chronic obstructive pulmonary disease, features pulmonary vascular remodeling driven by dysregulated pulmonary arterial smooth muscle cell (PASMC) apoptosis, with limited therapies like vasodilators offering only modest hemodynamic benefits. This study aimed to elucidate the role of Midkine (MK) in regulating PASMC apoptosis via the Notch2 pathway and its potential as a novel anti-remodeling target in HPH progression.

**Methods:**

The experimental approach involved cellular and animal models. *In vitro*, PASMCs were exposed to hypoxia (1% O_2_, 48 h), and MK was silenced via lentiviral shRNA (shRNA-413, ∼85% knockdown). Cellular phenotypes were assessed using Cell Counting Kit-8 assays for proliferation, wound healing assays for migration, and flow cytometry for apoptosis. Molecular analyses (quantitative polymerase chain reaction and western blot) targeted apoptosis-related proteins and Notch2 signaling. *In vivo*, HPH was induced in rats by LPS plus chronic hypoxia/smoke exposure; MK was inhibited via intratracheal adeno-associated virus serotype 9-mediated gene delivery (AAV9-shRNA (1.5 × 10^11^ vg/rat). Outcomes included hemodynamics, vascular remodeling, oxidative stress markers, and apoptosis-related protein expression in lung tissue.

**Results:**

*In vitro*, hypoxia upregulated MK, activating Notch2-Hes1 signaling, promoting proliferation and migration at 48 h, and suppressing apoptosis (*P* < 0.01 vs. normoxia). MK knockdown reversed these effects, restoring apoptosis (*P* < 0.01). *In vivo*, HPH activated the MK-Notch2 axis in rat lung tissue; MK suppression inhibited Notch2 signaling, improved antioxidant capacity, alleviated oxidative stress (e.g., elevated SOD/CAT, reduced MDA/LDH; *P* < 0.01), induced PASMC apoptosis, attenuated vascular remodeling, and reduced pulmonary arterial pressure.

**Conclusion:**

Unlike current vasodilators, MK drives HPH pathogenesis by suppressing PASMC apoptosis via Notch2 and exacerbating oxidative stress. Targeting MK offers a promising therapeutic strategy for HPH.

## 1 Introduction

Hypoxic pulmonary hypertension (HPH) is a debilitating clinical syndrome characterized by exertional dyspnea and fatigue, often arising as a complication of chronic obstructive pulmonary disease (COPD) (Mekov et al., 2025). Epidemiological data indicate that up to 91% of patients with advanced COPD may develop HPH (Pulmonary vascular disease group of chinese thoracic society, 2021). The hallmark pathology is pulmonary vascular remodeling, driven by dysregulated proliferation and impaired apoptosis of pulmonary arterial smooth muscle cells (PASMCs), alongside increased oxidative stress. Hypoxia-induced mitochondrial dysfunction in lung tissue promotes excessive PASMC proliferation and accelerates vascular remodeling (Shi et al., 2023). The mitochondrial apoptotic pathway plays a critical role in this process, as demonstrated in HPH rat models by a downregulated Bax/Bcl-2 ratio, impaired apoptosome assembly, and decreased cleaved caspase-3 expression, collectively suppressing PASMC apoptosis. These pathological changes narrow the pulmonary arterial lumen, elevate pulmonary arterial pressure (PAP), and may ultimately progress to right heart failure and death (Chen et al., 2021; Chi et al., 2022; Cao et al., 2019).

Despite advances in elucidating HPH mechanisms, the precise drivers of vascular remodeling remain incompletely understood, limiting therapeutic advancements. Current treatments, including phosphodiesterase-5 inhibitors and endothelin receptor antagonists (ERAs), modestly improve hemodynamics but offer limited gains in exercise capacity and overall quality of life (Boucly et al., 2025; Papadopoulos et al., 2025). Thus, identifying the pathogenic mechanisms of HPH and developing novel targeted strategies to inhibit PASMC hyperproliferation and remodeling is an urgent clinical priority.

Midkine (MK), a heparin-binding growth factor, plays key roles in inflammation and cell cycle regulation. It is highly expressed during embryogenesis to guide vascular and pulmonary development (Yıldırım et al., 2025). In adults, MK is minimally expressed but robustly upregulated by certain pathological states, including hypoxia. The Notch2 receptor, a key MK effector, modulates these processes and regulates cell proliferation and apoptosis via downstream targets like Hes1. The Notch signaling pathway exhibits high context-dependency in vascular remodeling (Zhang et al., 2023; Wang et al., 2023). In tumors, Notch2 activation often promotes cell proliferation and drug resistance ^[12]^, whereas in the cardiovascular system, the role of Notch is more complex (Zhang et al., 2023; Wang et al., 2023). Notably, the constitutively active Notch2 Intracellular Domain (NICD) can bypass ligand regulation to directly drive Hes1 expression (Zhang et al., 2023; Wang et al., 2023), a characteristic that has been widely utilized to establish the upstream regulatory relationships of Notch2 (Wang et al., 2023). The Notch2 receptor requires cleavage by γ-secretase to release its intracellular domain (NICD) for downstream signal activation (Zhang et al., 2023; Wang et al., 2023; Singh et al., 2023). *In vitro* studies demonstrated that hypoxic microenvironments induce MK overexpression, which activates the Notch2 downstream effector Hes1. This activation inhibits caspase-3 via the mitochondrial pathway, suppressing apoptosis and promoting aberrant proliferation of A549 non-small cell lung cancer cells (Zheng and Gao, 2019; Semenza, 2013; Aller et al., 2025). These effects are reversed by MK knockdown with siRNA, which enhances apoptosis and impedes tumor growth (Shin et al., 2020; Li et al., 2023). Aligning with this, our previous studies using COPD rat models showed hypoxia-induced upregulation of MK, Notch2 transmembrane form (NTM), and Hes1 in airway tissues. By reducing NTM and Hes1 levels, MK inhibition selectively induced apoptosis in damaged airway smooth muscle cells (ASMCs) without affecting healthy ones, thereby ameliorating airway remodeling. Importantly, MK suppression had no effect on normal ASMCs but specifically promoted apoptosis in damaged ASMCs (Deng et al., 2022; Sun et al., 2022). Hypoxia, LPS, and cigarette smoke all contribute to MK induction through distinct but convergent mechanisms: hypoxia via HIF-1α-dependent transcription (Reynolds et al., 2004; Daisuke et al., 2020), LPS via inflammatory cytokine upregulation (Tanino et al., 2025), and cigarette smoke via NF-κB-mediated oxidative stress (Paulina and Rafal, 2024). In our COPD-HPH model combining these stimuli, they likely act synergistically to enhance MK expression.

**FIGURE 1:**
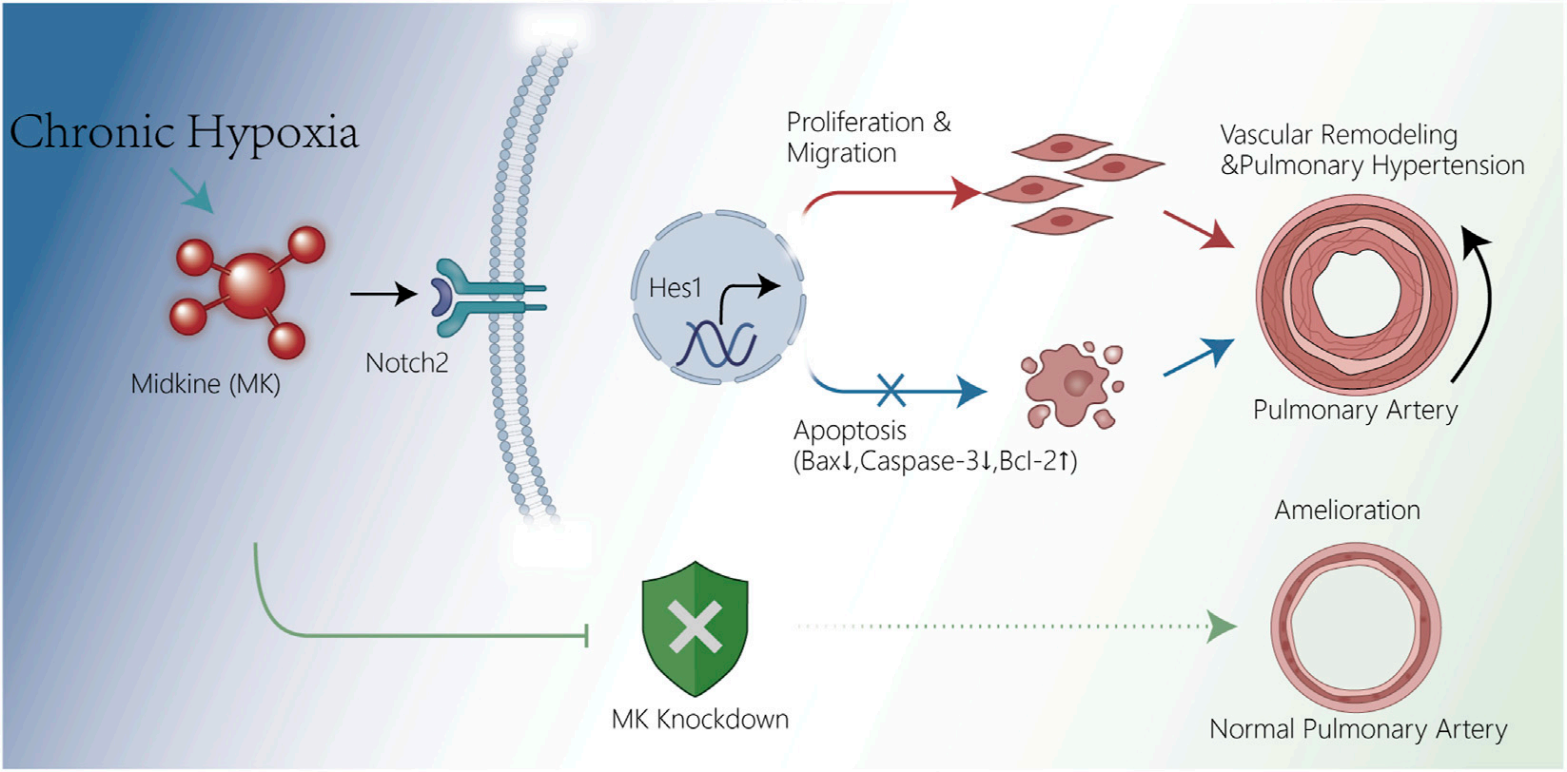
Schematic Overview of the MK-Notch2 Signaling Axis in HPH Pathogenesis and Therapeutic Targeting. Targeted inhibition of MK suppresses Notch2 signaling activation, thereby reducing oxidative stress and inducing apoptosis in PASMCs, alleviating pulmonary vascular remodeling, and lowering pulmonary arterial pressure in HPH. HPH, hypoxic pulmonary hypertension; MK, Midkine; PASMCs, pulmonary arterial smooth muscle cells.

Given that COPD is the most common etiology of HPH, we hypothesize that the MK-Notch2 signaling axis inhibits PASMC apoptosis and promotes excessive proliferation under hypoxic conditions. Consequently, targeted MK inhibition could offer a therapeutic avenue for ameliorating pulmonary vascular remodeling. In this study, we employed lentivirus-delivered shRNA to suppress MK expression in PASMCs under hypoxia *in vitro*. Furthermore, we administered AAV9-mediated MK shRNA via airway delivery in a rat model of COPD-associated HPH to investigate the mechanism underlying MK inhibition as a potential treatment for HPH. By elucidating the mechanistic contributions of MK-Notch2 signaling to HPH pathogenesis, this research aims to provide novel insights on MK inhibition as a therapeutic strategy for preventing the progressive increase in PAP in patients with HPH. A schematic overview of our mechanistic hypothesis is presented in Figure 1.

## 2 Methods

### 2.1 Animal model establishment and grouping

Animal housing conditions, experimental groups and treatments, and model validation are described in the Supplementary Materials.

### 2.2 Cell culture and transfection

Cell culture conditions, experimental groups and treatments, and corresponding product catalog numbers are described in the Supplementary Materials.

### 2.3 Selection of MK-Targeting shRNA and AAV9 vector preparation

Lentiviral shRNAs targeting MK were designed and cloned under a U6 promote, while recombinant AAV9 vectors expressing the selected MK shRNA were constructed with a cytomegalovirus promoter driving a GFP reporter for transduction tracking. Vectors were packaged, titered and provided by Shanghai GeneChem Co., Ltd. *In vitro* screening (as in Section 2.2) identified shRNA-413 as the most efficient which was cloned into the AAV9 backbone. Vector titers were confirmed by quantitative real-time PCR (qPCR). ShRNA sequences, vector maps, and construction protocols are provided in Supplementary Tables 1–5.

### 2.4 Cell migration assay

PASMCs (5 × 10^5^ cells/well) were seeded in 6-well plates and grown to 90%–100% confluence. A sterile 200 μL pipette tip was used to create a linear scratch wound. Cells were washed with phosphate-buffered saline (PBS) and cultured in serum-reduced medium (DMEM + 3% FBS). Under group-specific conditions (normoxia or hypoxia), migration was imaged at 0, 24, and 48 h using an inverted microscope (Olympus IX73). Wound closure was quantified as the percentage reduction in wound width using ImageJ software (version 1.53; NIH), with three random fields per well (n = 3 independent experiments).

### 2.5 Rescue experiments for MK-Notch2 epistasis analysis

To validate that MK is located upstream of the Notch2-Hes1 signaling axis, the following four experimental groups were established: CONT (normoxia), Hypoxia (hypoxia alone), Hypoxia + shMK (MK knockdown), and Hypoxia + shMK + NICD (MK knockdown + NICD overexpression). Cell proliferation was assessed by CCK-8 assay; apoptosis was detected by flow cytometry; and the expression of NICD, Hes1, and apoptosis-related proteins (Bax, Bcl-2, cleaved caspase-3) was determined by western blot. mRNA levels of MK, Notch2, and Hes1 were measured by qPCR. The efficiency of NICD overexpression was verified using an Anti-FLAG antibody (Abcam, ab205606).

### 2.6 Cell proliferation and apoptosis assay

The different groups of PASMCs (3 × 10^3^ cells/well) were cultured in triplicate in 96-well plates, and their proliferation was assessed by the CCK-8 assay. Furthermore, PASMCs (5 × 10^5^ cells/well) were cultured in six replicates in 6-well plates for 24 h. The cells were harvested and stained with 10 µL PI and 5 µL Annexin V-FITC in the dark, followed by flow cytometry analysis of the frequency of apoptotic cells.

### 2.7 *In Vivo* hemodynamic and vascular remodeling assessment

Four weeks post-AAV9 administration, rats were anesthetized (sodium pentobarbital, 40 mg/kg i. p.), and mPAP was measured via a PE-50 catheter inserted into the right jugular vein and advanced to the pulmonary artery, connected to a pressure transducer (PowerLab, ADInstruments). Heart rate and systemic pressure were monitored simultaneously. For vascular remodeling, intrapulmonary arteries (50–100 μm diameter) from formalin-fixed lungs were sectioned (5 μm), and the wall thickness percentage (WT%) and wall area percentage (WA%) were calculated using ImageJ after hematoxylin and eosin (H&E) staining. Verhoeff-Van Gieson elastic staining (EVG) staining was performed on parallel sections for qualitative assessment of elastic fiber integrity (n = 8–10 arteries/rat, three rats/group).

### 2.8 H&E staining

Rat lung tissues were fixed in 4% paraformaldehyde for 24 h, dehydrated through graded ethanol, embedded in paraffin, and sectioned at 5 μm. Sections were deparaffinized, rehydrated, stained with H&E (hematoxylin [5 min] and eosin [2 min]; Biosharp, Hefei, China) for histological evaluation, differentiated in 1% HCl-ethanol, and mounted. Images were captured using a light microscope (Olympus BX53) for qualitative assessment of vascular wall thickening and muscularization. Detailed EVG staining protocols for vascular tissues are provided in the Supplementary Materials.

### 2.9 Immunofluorescence (IF)

Frozen lung sections (5 μm) were air-dried, fixed in cold acetone (10 min), permeabilized with 0.3% Triton X-100 (30 min), and blocked with 4% goat serum (30 min). Sections were incubated overnight at 4 °C with primary antibody against GFP (1:500; Abcam, ab13970). Nuclei were counterstained with 4′,6-diamidino-2-phenylindole (DAPI; 1:2000; Abcam). Fluorescence images were acquired using an upright fluorescence microscope (OLYMPUS BX53). Detailed antibody dilutions and catalog numbers are provided in the Supplementary Materials.

### 2.10 Oxidative stress assays

Blood samples were collected from the abdominal aorta under anesthesia, and serum was isolated by centrifugation (3,000× g, 10 min at 4 °C). Following the manufacturer’s protocols, levels of superoxide dismutase (SOD), catalase (CAT), MDA, and LDH were quantified in serum using commercial colorimetric assay kits (Nanjing Jiancheng Bioengineering Institute, China), with serum samples assayed directly. For BALF and tissue homogenates, results were normalized to the total protein content (bicinchoninic acid [BCA] assay; Pierce) and expressed as units per milligram protein (U/mgprot) or nanomoles per milligram protein (nmol/mgprot) (n = 3 rats/group).

### 2.11 Western blotting

Protein was extracted from PASMCs or lung tissues using radioimmunoprecipitation assay lysis buffer. Protein concentration was determined by BCA assay, and equal amounts (10 μg) were separated by sodium dodecyl sulfate polyacrylamide gel electrophoresis and transferred to polyvinylidene fluoride membranes. After blocking with 5% non-fat milk, membranes were incubated overnight at 4 °C with the indicated primary antibodies, followed by incubation with HRP-conjugated secondary antibodies (1:5000) for 1 h. Protein bands were visualized by enhanced chemiluminescence, and band intensity was quantified using ImageJ software, normalized to β-actin or tubulin. Experiments were performed in triplicate. Detailed antibody dilutions and catalog numbers are provided in the Supplementary Materials.

### 2.12 qPCR

Total RNA was extracted from PASMCs or lung tissues using TRIzol reagent (Promega) and reverse-transcribed from 1 μg RNA with a PrimeScript RT Reagent Kit (Takara). qPCR was performed in 20 μL reactions containing SYBR Green Master Mix (Promega) on a QuantStudio 5 system, using the following cycling conditions: 95 °C for 30 s, followed by 40 cycles of 95 °C for 5 s and 60 °C for 30 s. Primers for MK, Notch2, Hes1, and β-actin were designed and synthesized by Sangon Biotech. Gene expression was analyzed using the 2^−ΔΔCT^ method normalized to β-actin (n = 3). Primer sequences are provided in Supplementary Table 3.

### 2.13 Statistical analysis

Data are presented as mean ± standard deviation (SD). GraphPad Prism software (version 10.0; GraphPad Software, San Diego, CA, United States) was used for analysis. For multiple comparisons, one-way analysis of variance (ANOVA) followed by Tukey’s *post hoc* test was applied. A *p*-value < 0.05 was considered statistically significant. Significance levels are denoted as follows: ns (not significant), *P < 0.05, **P < 0.01.

## 3 Results

### 3.1 Screening of MK-Targeting shRNAs and Validation of Knockdown Efficiency

To identify an effective RNA interference tool against MK, we screened three lentiviral shRNA constructs (shRNA-411, shRNA-412, shRNA-413) in PASMCs, alongside non-targeting scrambled shRNA (Non-target) and untreated controls (CONT). Transduction was performed under normoxic conditions, enhanced by HitransG P or A reagents (GeneChem).

**FIGURE 2:**
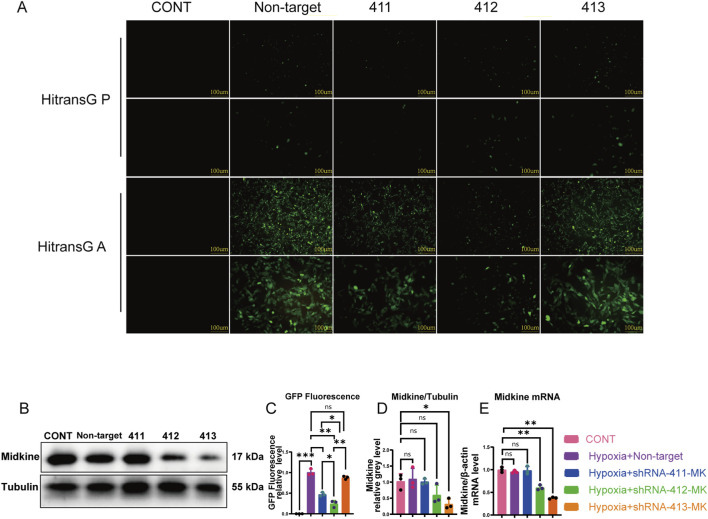
Screening of Midkine-Targeting shRNAs and Validation of Knockdown Efficiency. **(A)** Fluorescence microscopy images showing GFP expression in PASMCs 72 h post-transduction under normoxic conditions, treated with HitransG P or A enhancers, non-targeting shRNA (Non-target), or MK-specific shRNAs (shRNA-411, shRNA-412, shRNA-413). Scale bar: 200 μm. **(B,D)** Representative western blot images **(B)** and densitometric quantification **(D)** of MK protein levels normalized to β-actin. **(C)** Quantitative analysis of GFP-positive cell percentage from fluorescence imaging. Data are presented as mean ± SD (n = 3 independent experiments). **(E)** Relative MK mRNA expression determined by qPCR. *P < 0.05, **P < 0.01 vs. CONT or indicated groups (one-way ANOVA with Tukey’s *post hoc* test). ANOVA, analysis of variance; CONT, control; GFP, green fluorescent protein; MK, Midkine; PASMCs, pulmonary artery smooth muscle cells; SD, standard deviation; shRNAs, short hairpin RNAs.

At 72 h post-transduction, transduction efficiency was assessed by GFP expression via fluorescence microscopy. As shown in Figure 2A, the CONT group showed no GFP signal, while the Non-target group exhibited robust fluorescence (>90% positive cells), confirming high viral uptake. All MK-targeting shRNAs achieved efficient transduction, with shRNA-413 displaying the strongest GFP intensity, indicative of optimal infection. HitransG A outperformed HitransG P in enhancing transduction across groups (quantitative analysis in Figure 2D; *P* < 0.05).

Knockdown efficiency at the protein level was evaluated by western blot. Representative immunoblots (Figure 2B) and densitometric quantification (Figure 2C) demonstrated that all three shRNAs significantly reduced MK expression relative to CONT (*P* < 0.01 vs. CONT), with no change in the Non-target group (not significant). ShRNA-413 achieved the greatest reduction (85% knockdown; *P* < 0.001 vs. other shRNAs and CONT), making it the most effective. To validate shRNA efficiency, qPCR confirmed that shRNA-413 reduced MK mRNA by ∼62% (*P* < 0.01), consistent with western blot showing ∼85% protein knockdown (Figures 2B–E). In contrast, shRNA-411 and -412 showed no significant mRNA reduction (*P* > 0.05). Based on dual validation of qPCR, western blot, and GFP fluorescence, shRNA-413 was selected for subsequent functional studies.

### 3.2 Silencing of MK suppresses hypoxia-induced migration and proliferation of PASMCs and reverses apoptosis resistance

To assess the functional impact of MK knockdown, we examined migration, proliferation, and apoptosis in PASMCs under hypoxic conditions using the selected shRNA-413 (Hypoxia + shRNA-MK group) compared to CONT.

**FIGURE 3:**
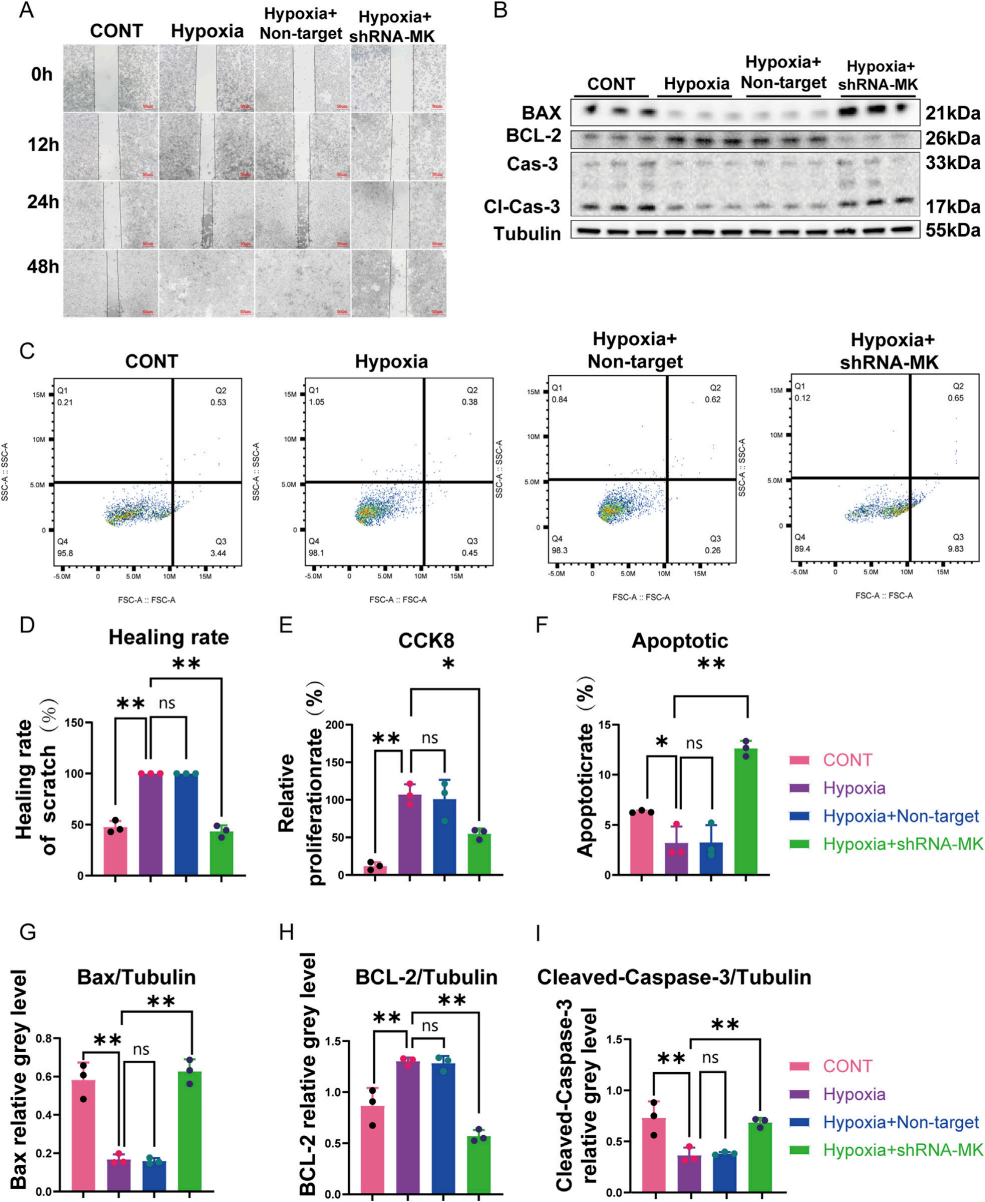
*In Vitro* Functional Effects of MK Knockdown in Hypoxic PASMCs. **(A,D)** Representative images **(A)** and quantitative analysis **(D)** of wound healing assay showing migration at 0, 24, and 48 h across groups: CONT (normoxia), Hypoxia (1% O_2_), Hypoxia + Non-target control (non-target shRNA lentivirus, designed not to target any known mammalian genes, including MK, to control for nonspecific effects of the lentiviral vector), Hypoxia + shRNA-MK (MK-targeting shRNA lentivirus). Wound closure is expressed as percentage relative to 0 h. Scale bar: 500 μm. **(B, G–I)** Representative western blots **(B)** and densitometric quantification **(G–I)** of Bax, Bcl-2, and cleaved caspase-3 levels normalized to β-actin. **(C,F)** Flow cytometry plots **(C)** and quantification **(F)** of apoptosis (Annexin V+/PI + cells) after 48 h **(E)** CCK-8 proliferation assay. Data are presented as mean ± SD (n = 3 independent experiments). *P < 0.05, **P < 0.01 vs. CONT or indicated groups (one-way ANOVA with Tukey’s *post hoc* test). ANOVA, analysis of variance; CCK-8, Cell Counting Kit-8; CONT, control; MK, Midkine; PASMCs, pulmonary artery smooth muscle cells; SD, standard deviation; shRNA, short hairpin RNA.

Wound healing assays revealed that hypoxia significantly enhanced PASMC migration. As shown in representative images (Figure 3A) and quantitative analysis (Figure 3D), the Hypoxia and Hypoxia + Non-target groups displayed rapid wound closure, whereas MK knockdown markedly inhibited this effect (*P* < 0.01 vs. hypoxia). Western blot analysis of mitochondrial apoptotic pathway regulators (Figures 3B,G–I) demonstrated that hypoxia suppressed pro-apoptotic Bax and cleaved caspase-3 while elevating anti-apoptotic Bcl-2 (*P* < 0.01 vs. CONT). MK knockdown reversed these changes, increasing Bax and cleaved caspase-3 (*P* < 0.01) and decreasing Bcl-2 (*P* < 0.01). Flow cytometry with Annexin V/PI staining (Figures 3C,F) confirmed that hypoxia reduced apoptosis (*P* < 0.05 vs. CONT), an effect unchanged by non-target shRNA but restored by MK silencing (*P* < 0.01 vs. Hypoxia). The CCK-8 assay (2 h incubation) further showed hypoxia-induced proliferation (Figure 3E; ∼1.8-fold increase; *P* < 0.01 vs. CONT), which was abolished by MK knockdown (comparable to CONT; *P* < 0.05 vs. hypoxia).

Collectively, these data indicate that MK silencing inhibits hypoxia-driven migration and proliferation while sensitizing PASMCs to apoptosis, underscoring MK’s role in the pathological phenotype.

### 3.3 Silencing of MK in hypoxia-exposed PASMCs attenuates the Notch2-Hes1 signaling axis

**FIGURE 4:**
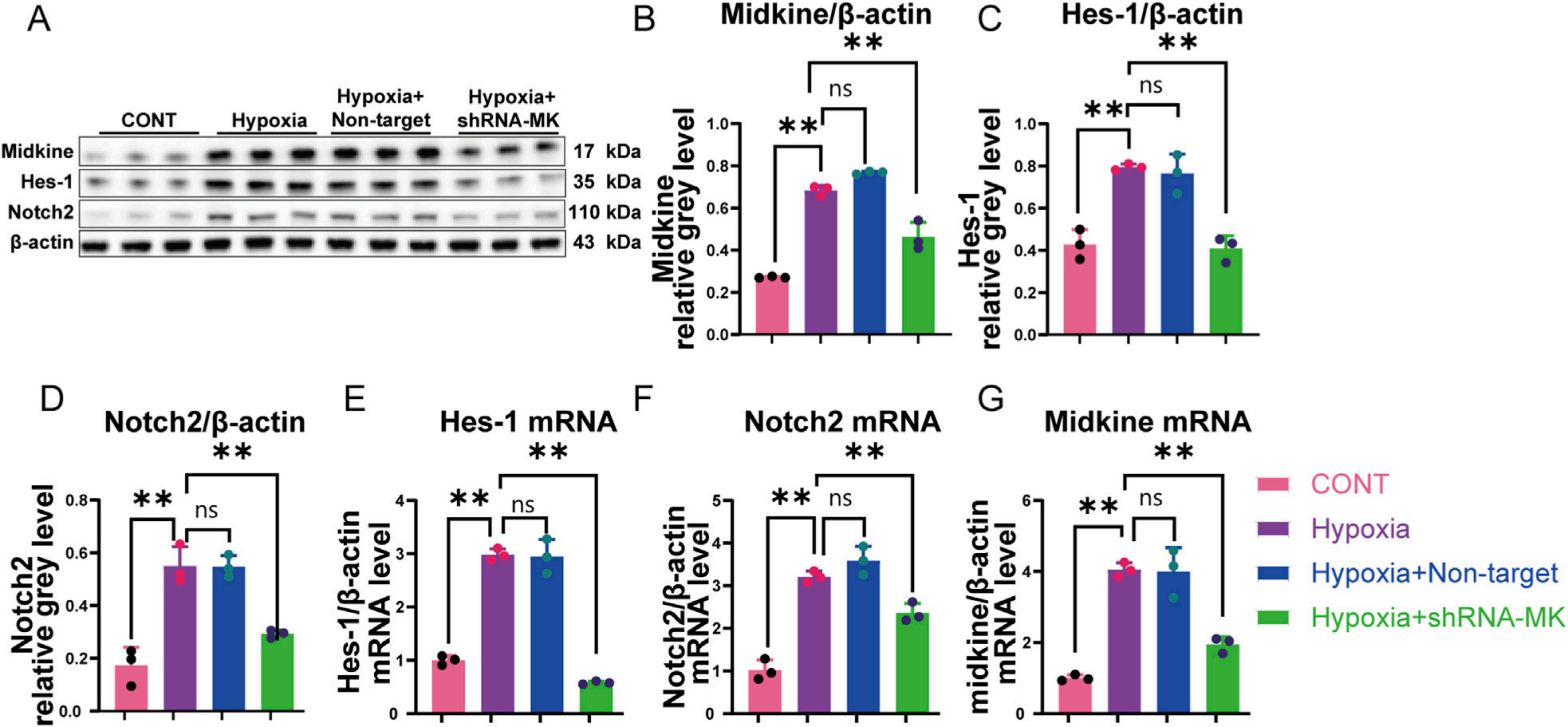
MK Knockdown Suppresses Notch2-Hes1 Signaling in Hypoxic PASMCs. **(A–D)** Representative western blots **(A)** and densitometric quantification **(B–D)** of MK, Notch2, and Hes1 protein levels normalized to β-actin after 48 h under group conditions. **(E–G)** qPCR analysis of relative mRNA expression (2^−ΔΔCT^ method, normalized to β-actin) for MK, Notch2, and Hes1. Data are presented as mean ± SD (n = 3 independent experiments). *P < 0.05, **P < 0.01 vs. CONT or indicated groups (one-way ANOVA with Tukey’s *post hoc* test). ANOVA, analysis of variance; CONT, control; MK, Midkine; PASMCs, pulmonary artery smooth muscle cells; qPCR, quantitative polymerase chain reaction.

Prior studies in COPD models showed that MK suppression downregulates Notch2-Hes1 signaling and promotes ASMC apoptosis (Semenza, 2013; Aller et al., 2025). Given that COPD is a predominant cause of HPH, we hypothesized that MK knockdown in PASMCs under hypoxic conditions would similarly inhibit Notch2-Hes1 signaling. Western blot and qPCR analyses revealed that hypoxia markedly activates the MK-Notch2 signaling axis, significantly upregulating MK, Notch2, and its downstream effector Hes1 at protein levels (Figures 4A–D; ∼2–3-fold increase; *P* < 0.01 vs. CONT) and mRNA levels (Figures 4E–G; ∼3–4-fold; *P* < 0.01 vs. CONT). Non-target shRNA had no effect (not significant), but MK knockdown (Hypoxia + shRNA-MK) substantially attenuated this activation, reducing protein and mRNA expression to near-CONT levels (*P* < 0.01 v. Hypoxia). These findings suggest that MK plays a pivotal role in modulating the Notch2-Hes1 signaling pathway under hypoxic conditions, thereby influencing the biological functions of PASMCs.

### 3.4 Epistasis analysis confirms MK as an upstream regulator of Notch2

Epistasis analysis confirmed that NICD overexpression in MK-silenced cells (85% reduction) restored Hes1 (*P* < 0.05) and partially rescued proliferation/apoptosis (Figure 9), supporting a model in which MK functions upstream of Notch2-Hes1 in modulating these cellular phenotypes, though we cannot exclude contributions from parallel or intersecting pathways.

### 3.5 Targeted inhibition of MK ameliorates pulmonary vascular remodeling in HPH

**FIGURE 5:**
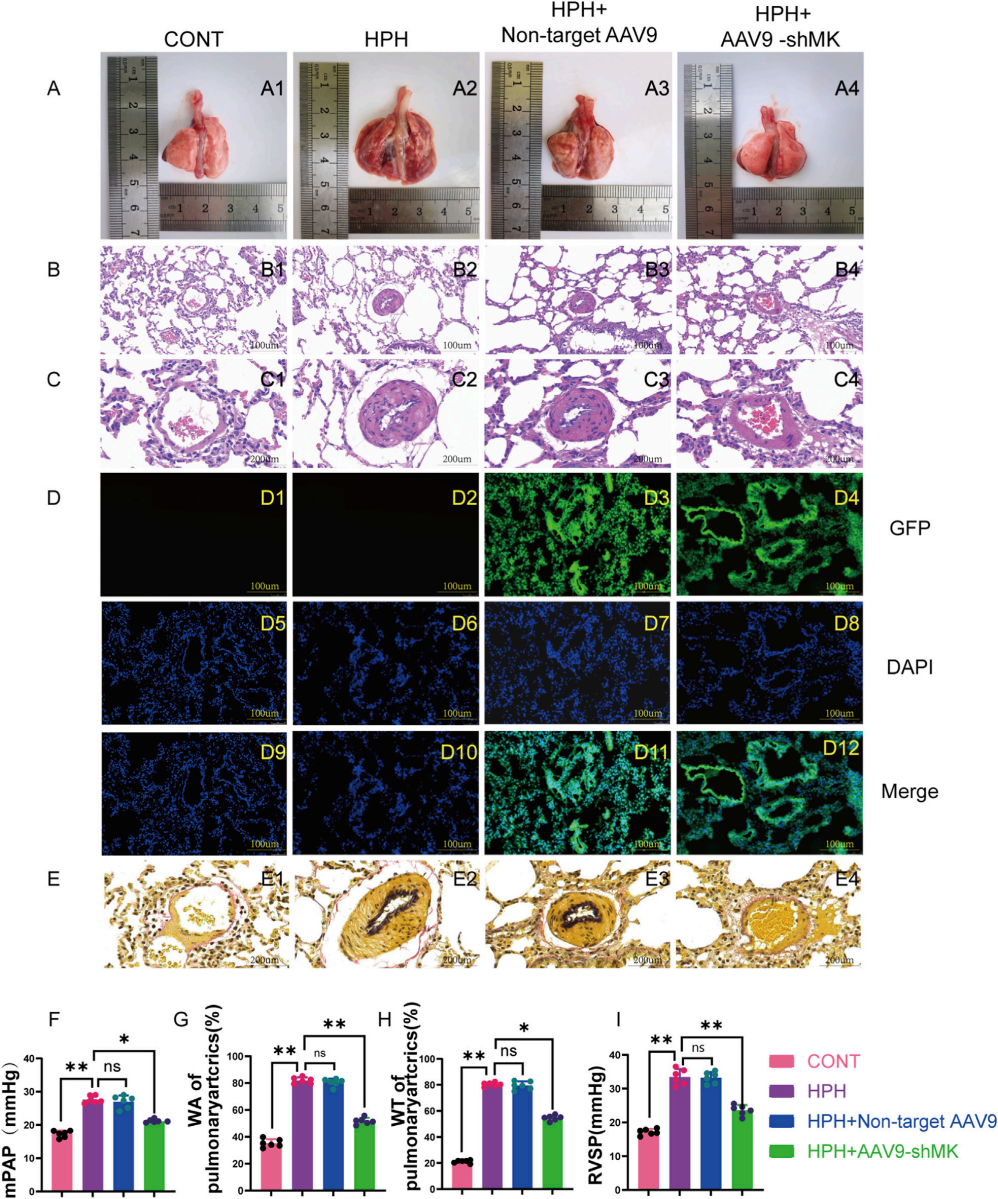
*In Vivo* Effects of Targeted MK Knockdown on Pulmonary Vascular Remodeling in HPH. **(A)** Gross morphology of lung tissues from CONT, HPH, HPH + Non-target AAV9, and HPH + AAV9-MK groups. **(B,C)** Representative H&E-stained pulmonary artery sections **(B)**; low magnification, scale bar: 200 μm) and higher-magnification views **(C)**; scale bar: 100 μm) showing wall thickening and inflammation. **(D)** Immunofluorescence images of GFP (green) in frozen lung sections with DAPI counterstain (blue; scale bar: 100 μm). **(E)** EVG-stained sections highlighting elastic fibers (black) and collagen (red; scale bar: 200 μm). **(F–H)** Quantification of mPAP **(F)**, wall thickness percentage (WT%; **(G)**, and wall area percentage (WA%; **(H)**. WT% = (medial wall thickness/external diameter) ×100; WA% = (wall area/total vessel area) ×100. Data are presented as mean ± SD (n = 6 rats/group; 8–10 arteries/rat). **(I)** Quantitative analysis of right ventricular systolic pressure (RVSP). *P < 0.05, **P < 0.01 vs. CONT or indicated groups (one-way ANOVA with Tukey’s *post hoc* test). ANOVA, analysis of variance; MK, Midkine; CONT, control; AAV9, adeno-associated virus serotype 9; H&E, hematoxylin and eosin; HPH, hypoxic pulmonary hypertension; GFP, green fluorescent protein; DAPI, 4′,6-diamidino-2-phenylindole; EVG, elastic Van Gieson; mPAP, mean pulmonary arterial pressure; SD, standard deviation; WT%, wall thickness percentage; WA%, wall area percentage.

To evaluate the therapeutic efficacy of MK inhibition in HPH, we delivered AAV9-shRNA targeting MK (or non-target CONT) intratracheally to HPH rats and assessed lung morphology, histopathology, vascular structure, and hemodynamics 21 days post-treatment. Gross lung examination (Figure 5A) showed marked inflammation, edema, congestion, and irregular surfaces in HPH and HPH + Non-target AAV9 groups compared to CONT, effects substantially alleviated by MK knockdown (HPH + AAV9-MK). H&E staining (Figures 5B,C) confirmed arterial wall thickening, luminal narrowing, and perivascular inflammation in HPH and non-target groups (*P* < 0.01 vs. CONT), which were reversed by MK inhibition (*P* < 0.05 vs. HPH). IF for GFP (Figure 5D) verified efficient AAV9 transduction (>70% GFP-positive cells in alveolar and vascular regions) in both vector-treated groups. EVG staining (Figure 5E) revealed disrupted elastic laminae, medial hypertrophy, and collagen deposition in HPH vessels, indicative of remodeling; these changes were mitigated by MK knockdown. Quantitative morphometry (Figures 5G,H) demonstrated elevated WT% and WA% in HPH and non-target groups (*P* < 0.01 vs. CONT), reduced by ∼30% in HPH + AAV9-MK (*P* < 0.01 vs. HPH). Hemodynamic assessment showed elevations of mPAP and RVSP in the HPH and non-target groups (Figures 5F,I; *P* < 0.01 vs. CONT), both of which were reduced by MK inhibition (*P* < 0.05 vs. HPH). These data indicate that MK knockdown attenuates vascular remodeling and lowers PAP, highlighting its therapeutic potential in HPH.

### 3.6 Targeted inhibition of MK promotes apoptosis in PASMCs via the mitochondrial pathway in an HPH model

**FIGURE 6:**
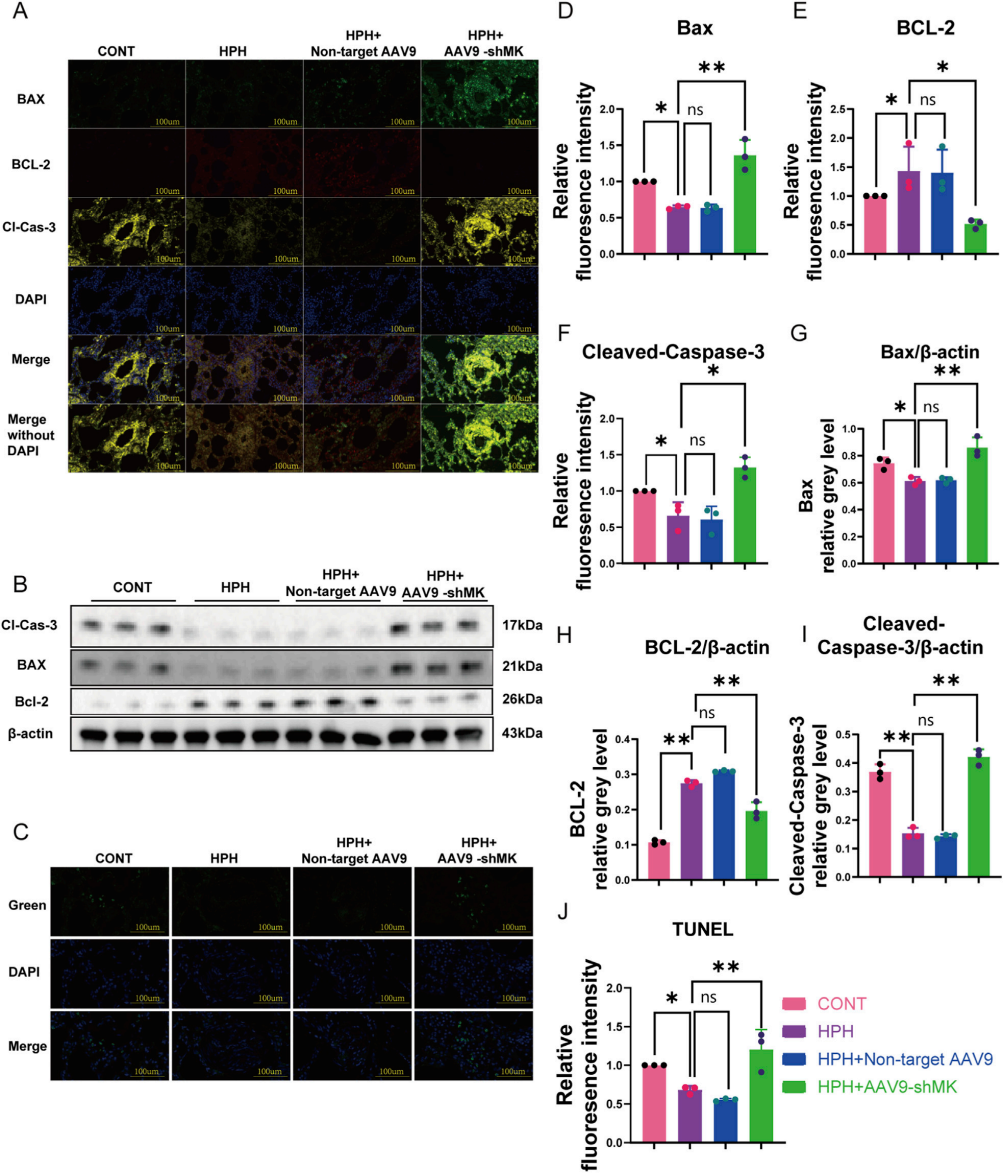
MK Knockdown Enhances PASMC Apoptosis via the Mitochondrial Pathway in HPH. **(A, D–F)** Representative immunofluorescence images **(A)** of pulmonary artery sections stained for Bax (green), Bcl-2 (red), and cleaved caspase-3 (green) with DAPI (blue), including merged views (scale bar: 100 μm), and quantitative fluorescence intensity (D–F; arbitrary units, normalized to area). **(B, G–I)** Western blots **(B)** and densitometric quantification **(G–I)** of Bax, Bcl-2, and cleaved caspase-3 in lung tissues, normalized to β-actin. **(C,J)** TUNEL-stained sections (C; green for apoptotic nuclei, DAPI blue; scale bar: 100 μm) and apoptotic index (J; TUNEL+/total nuclei ×100%). **(D–F, J)** Values normalized to the CONT group (set as 1). Data are presented as mean ± SD (n = 6 rats/group; five fields/section). *P < 0.05, **P < 0.01 vs. CONT or indicated groups (one-way ANOVA with Tukey’s *post hoc* test). ANOVA, analysis of variance; CONT, control; DAPI, 4′,6-diamidino-2-phenylindole; HPH, hypoxic pulmonary hypertension; MK, Midkine; PASMC, pulmonary artery smooth muscle cell; SD, standard deviation; TUNEL, terminal deoxynucleotidyl transferase dUTP nick end labeling.

We next examined whether MK knockdown enhances PASMC apoptosis *in vivo* through the mitochondrial pathway, using western blot, IF, and TUNEL assays on lung tissues from HPH rats. Western blot analysis (Figures 6B,G–I) showed that HPH downregulated pro-apoptotic Bax and cleaved caspase-3 (∼50% reduction; *P* < 0.01 vs. CONT) while upregulating anti-apoptotic Bcl-2 (∼2-fold; *P* < 0.01 vs. CONT). Non-target AAV9 had no effect (not significant), but MK knockdown restored Bax and cleaved caspase-3 (∼1.5–2-fold increase; *P* < 0.01 vs. HPH) while reducing Bcl-2 (∼60%; *P* < 0.01 vs. HPH). IF staining (Figures 6A,D–F) corroborated these shifts: diminished Bax and cleaved caspase-3 fluorescence in HPH vessels (*P* < 0.01 vs. CONT), unchanged by non-target AAV9, but intensified by MK inhibition (*P* < 0.01 vs. HPH); Bcl-2 signals followed the inverse pattern. TUNEL assay (Figures 6C,J) quantified apoptosis as ∼3% positive cells in HPH and non-target groups (*P* < 0.05 vs. CONT ∼5%), elevated to ∼11.8% in HPH + AAV9-MK (*P* < 0.01 vs. HPH). These findings demonstrate that MK inhibition reverses HPH-induced apoptosis resistance in PASMCs by activating the Bax/Bcl-2/caspase-3 pathway, contributing to reduced vascular remodeling with alleviated inflammation, edema, and elastic lamina disruption (Figures 5A–E).

### 3.7 *In Vivo* Validation of MK inhibition on downregulation of the Notch2-Hes1 pathway

**FIGURE 7:**
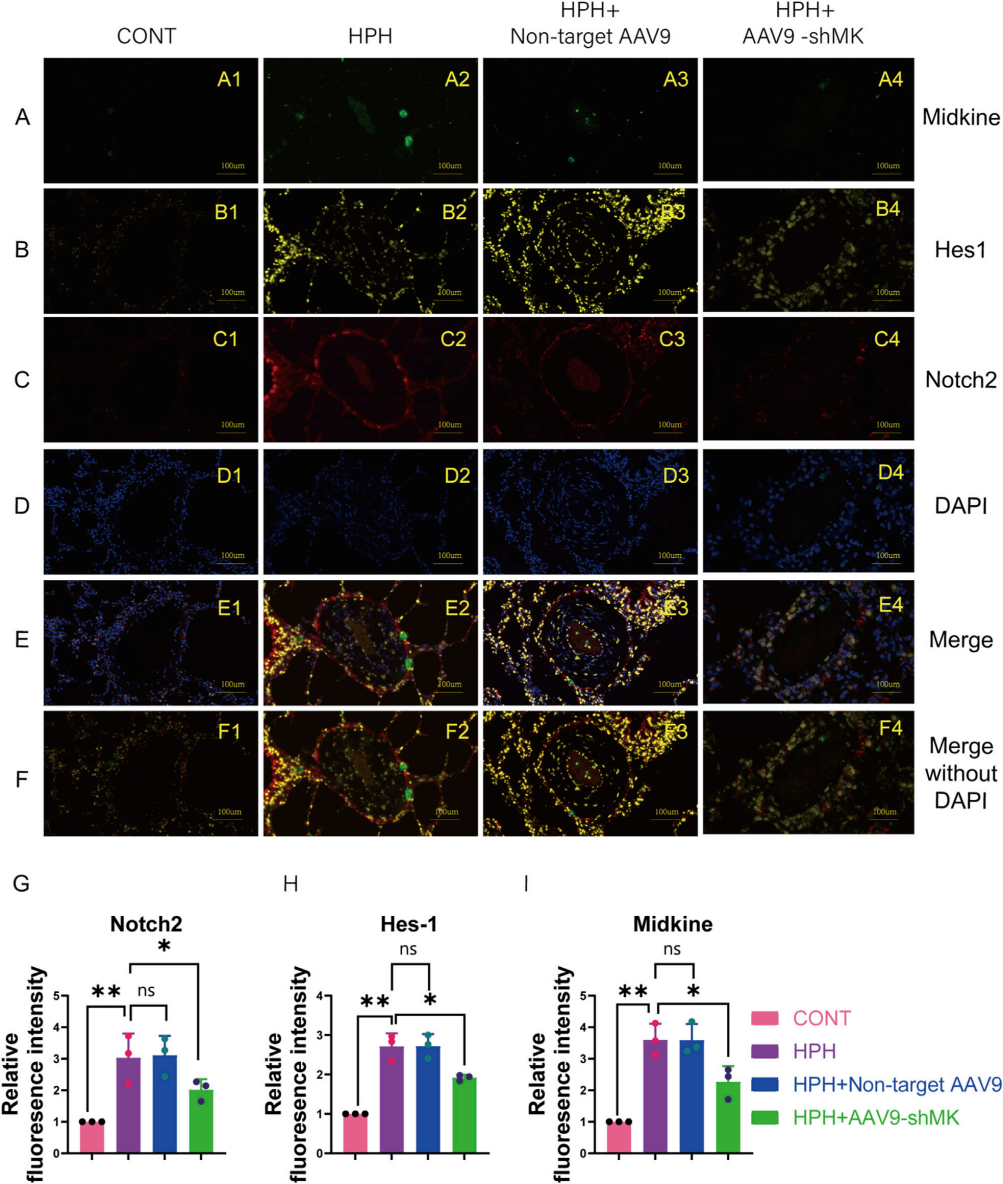
*In Vivo* Validation of MK-Mediated Regulation of the Notch2-Hes1 Pathway. **(A–F)** Immunofluorescence staining of pulmonary artery sections from rats subjected to different experimental interventions. Representative images (from top to bottom) illustrate the expression and localization of MK, Notch2, and Hes1 proteins. Included are views of DAPI-stained nuclei, merged fluorescence channels, and merged images without nuclear staining. **(G–I)** Quantitative analysis of immunofluorescence intensity for MK **(G)**, Notch2 **(H)**, and Hes1 **(I)** in pulmonary artery sections across the treatment groups. Data are presented as mean ± SD; n = 3 biologically independent samples. *P < 0.05, **P < 0.01. DAPI, 4′,6-diamidino-2-phenylindole; MK, Midkine; SD, standard deviation.

To further validate the regulatory role of MK on the Notch2-Hes1 signaling pathway under physiological conditions, we performed IF staining to examine the expression and localization of MK, Notch2, and Hes1 in pulmonary artery tissues from each experimental group. As illustrated in Figure 7, MK expression was markedly upregulated in the HPH group compared to the CONT group (*P* < 0.01), accompanied by significant activation of Notch2 and Hes1 proteins (*P* < 0.01). These findings indicate a substantial enhancement of the MK-Notch2-Hes1 signaling axis within the pulmonary arteries of HPH rats. No significant differences in protein expression were detected between the HPH group and the HPH + Non-target AAV9 group (ns), confirming that the empty viral vector did not influence pathway activity. In contrast, targeted MK inhibition in the HPH + AAV9-MK group resulted in a significant downregulation of Notch2 and Hes1 activation *P* < 0.05). Collectively, these *in vivo* results demonstrate robust activation of the MK-Notch2-Hes1 signaling pathway in HPH and show that AAV9-mediated MK suppression effectively attenuates this aberrant signaling, supporting MK’s role in promoting pulmonary vascular remodeling in HPH via the Notch2-Hes1 axis.

### 3.8 MK regulation of the Notch2-Hes1 pathway and enhances systemic antioxidant defense in vivo

**FIGURE 8:**
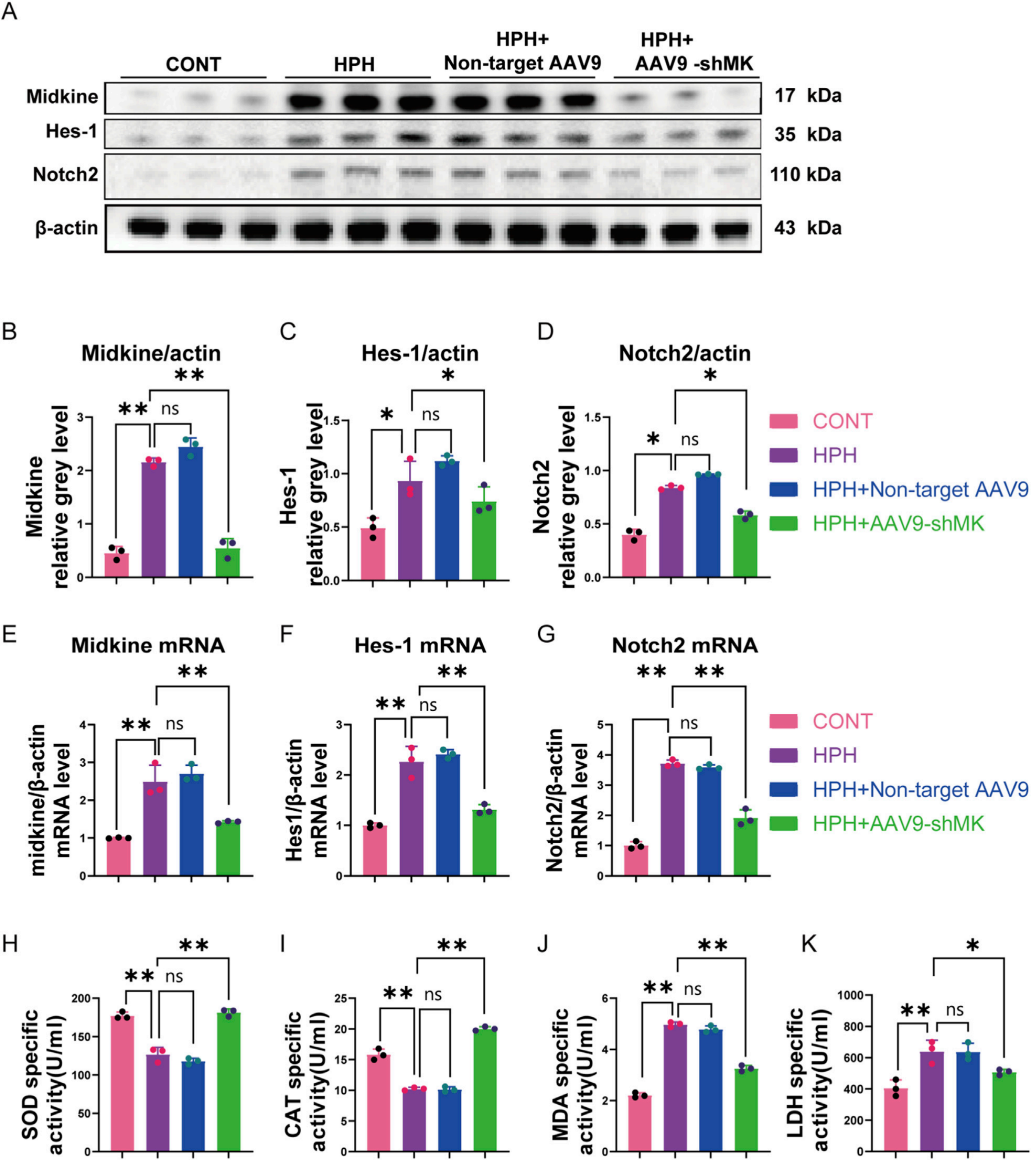
*In Vivo* Effects of MK Knockdown on Notch2-Hes1 Signaling and Oxidative Stress. **(A)** Representative western blots and **(B–D)** densitometric quantification of MK, Notch2, and Hes1 protein levels in lung tissues (normalized to β-actin). **(E–G)** Relative mRNA expression (2^−ΔΔCT^ method, β-actin) of MK, Notch2, and Hes1 by qPCR. **(H–K)** Serum oxidative stress markers (SOD, CAT, MDA, LDH) by colorimetric assay. Data are mean ± SD (n = 6 rats/group). *P < 0.05, **P < 0.01 vs. CONT (ANOVA with Tukey’s test). See above for abbreviations.

To further delineate the *in vivo* regulatory effect of MK inhibition on the Notch2-Hes1 signaling axis, we assessed MK, Notch2, and Hes1 expression at the protein and mRNA levels in rat lung tissues using western blot and qRT-PCR analyses. As demonstrated in Figures 8A–D, protein expression levels of MK, Notch2, and Hes1 were significantly elevated in the HPH and HPH + Non-target AAV9 groups compared to the CONT group (*P* < 0.05), with no statistically significant differences observed between these two groups (ns). In contrast, targeted MK knockdown (HPH + AAV9-MK group) markedly suppressed expression of all three proteins (*P* < 0.01), restoring levels comparable to those in the CONT group. Consistent with these protein findings, MK, Notch2, and Hes1 mRNA levels (Figures 8E–G) were significantly upregulated in the HPH and non-target AAV9 groups (*P* < 0.01) but were effectively reversed by MK knockdown. We next evaluated systemic oxidative stress levels using commercial biochemical assay kits (Figures 8H–K). Serum analysis revealed significantly reduced SOD and CAT activities (*P* < 0.01) and substantially elevated MDA and LDH levels (*P* < 0.01) in the HPH and non-target AAV9 groups compared to the CONT group, indicating impaired antioxidant capacity with no notable difference between these two groups (ns). AAV9-mediated MK silencing was associated with improved antioxidant profiles. In summary, targeted MK inhibition suppressed Notch2-Hes1 signaling and was associated with improved antioxidant enzyme activities and reduced lipid peroxidation markers in serum in HPH, highlighting MK’s potential involvement in both Notch2-Hes1 signaling and systemic antioxidant responses. It should be noted that LDH in serum primarily reflects cellular membrane damage and tissue injury rather than direct ROS generation. Its elevation in HPH likely indicates ongoing parenchymal and vascular damage, which may be secondary to oxidative stress.

**FIGURE 9:**
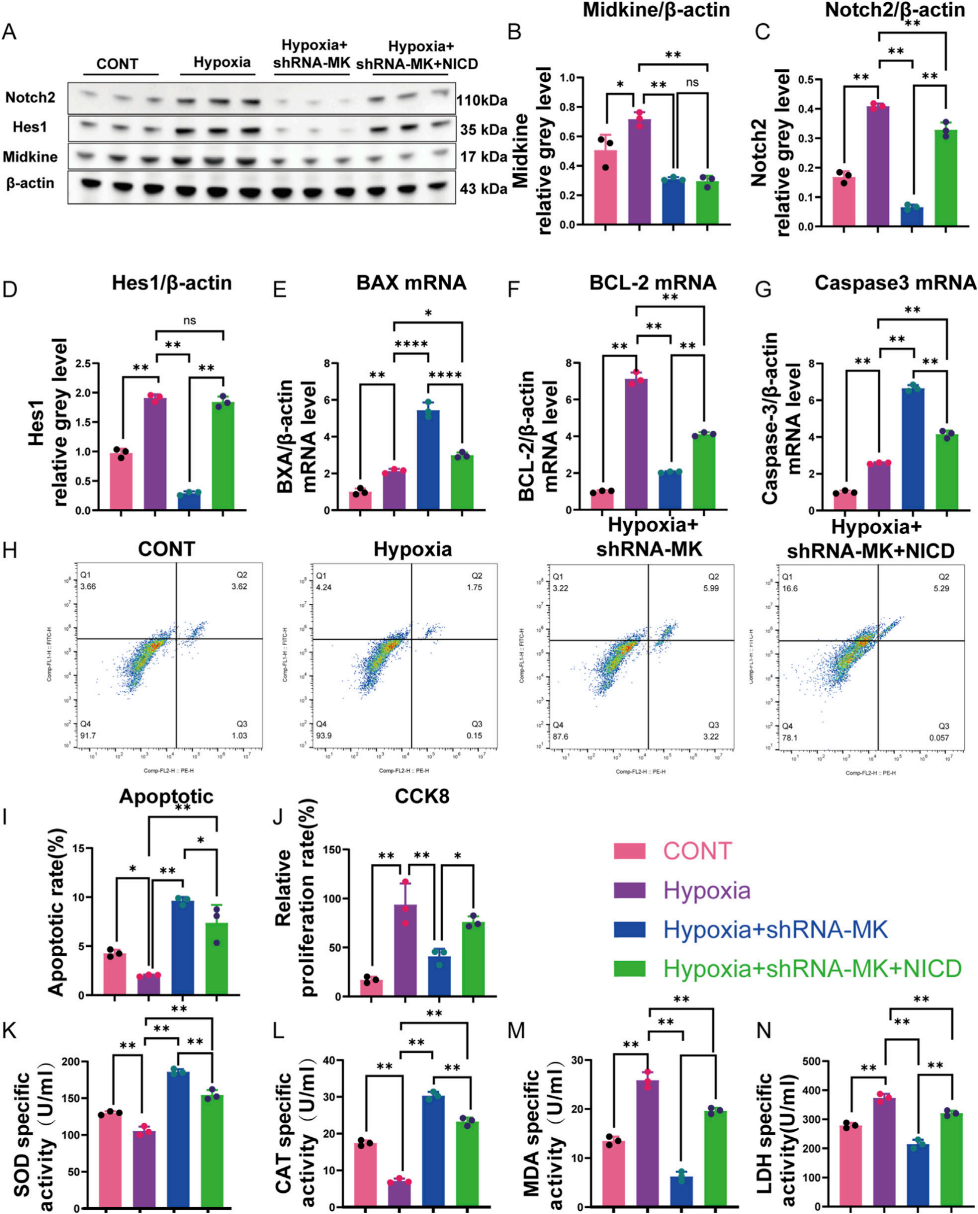
Epistasis analysis supports that MK acts upstream of the Notch2-Hes1 signaling pathway in hypoxic PASMCs. **(A)** Western blot results of MK, Notch2, NICD (Flag), Hes1 and β-actin; **(B–D)** Quantitative analysis of the expression of the above proteins; **(E–G)** qPCR analysis of mRNA expression of apoptosis-related genes Bax, Bcl-2 and Caspase-3; **(H–I)** Flow cytometry plots and quantitative analysis of cell apoptosis; **(J)** Cell proliferation/viability detected by the CCK-8 assay; **(K–N)** Detection results of oxidative stress markers SOD, CAT, MDA and LDH. All data are presented as mean ± standard deviation (n = 3). *P < 0.05, **P < 0.01, one-way analysis of variance (ANOVA) followed by Tukey’s post-hoc test versus the CONT group. See above for abbreviations.

## 4 Discussion

MK, a heparin-binding growth factor, is highly expressed during embryogenesis to orchestrate vascular and pulmonary development (Yıldırım et al., 2025; Tanino et al., 2025; Grabmaier et al., 2025). In healthy adults, MK expression is minimal, primarily in renal and intestinal epithelia, but it is reactivated under pathological conditions, contributing to inflammatory diseases. For instance, elevated serum MK in COVID-19 patients recruits macrophages and neutrophils to the alveoli, amplifying pro-inflammatory cytokines (TNF-α, IL-1β, IL-6, interferon-γ) and exacerbating respiratory distress (Sanino et al., 2020; Ketenci et al., 2022). HPH shares similar inflammatory drivers: hypoxia, via chemokine receptor CCR5, promotes IL-1β, IL-6, MIF, and HMGB1 accumulation (Tanino et al., 2025; Grabmaier et al., 2025; Sanino et al., 2020; Ketenci et al., 2022; Hu et al., 2020; Amsellem et al., 2014), fostering a milieu that inhibits PASMC apoptosis, enhances proliferation, and drives vascular remodeling—the hallmark of HPH. This remodeling narrows arterial lumina, elevates PAP, increases right ventricular afterload, and risks heart failure or death (Dai et al., 2025). Notably, COPD, the leading cause of HPH, overlaps in pathology with chronic inflammation, oxidative stress, and smooth muscle dysregulation. Our prior work established the MK-Notch2 signaling axis in COPD-induced airway remodeling (Deng et al., 2022; Sun et al., 2022). Mechanistically, the canonical Notch signaling cascade converges on the generation and nuclear translocation of the NICD, which subsequently binds to CSL/RBPjk to activate downstream transcriptional programs, including Hes1 (Ilagan et al., 2011). This process is subject to stringent spatiotemporal regulation. For instance, in vascular endothelial cells, RHOQ, a member of the Rho GTPase family, is induced by DLL4/Notch signaling and is essential for nuclear transport of NICD; loss of RHOQ results in cytoplasmic sequestration and autophagic degradation of NICD, thereby disrupting signal transduction (Bridges et al., 2020). Similarly, in cerebral ischemia, increased NICD expression in endothelial cells within the peri-infarct regions drives angiogenic remodeling (Ren et al., 2018). Using an LPS- and CS-induced COPD rat model, we selected HPH subsets (mPAP ≥25 mmHg) to dissect MK’s role in PASMC apoptosis resistance and remodeling. Our *in vitro* and *in vivo* data reveal MK as a central HPH driver: it activates Notch2-Hes1 signaling, suppresses mitochondrial apoptosis, and intensifies oxidative stress, collectively fueling remodeling. Targeted MK knockdown reversed these effects, positioning MK as a promising therapeutic target.

*In vitro*, hypoxia upregulated MK in PASMCs, activating Notch2 and its effector Hes1 (Figure 4), consistent with oncology models where hypoxic MK overexpression in A549 cells inhibits caspase-3-mediated apoptosis via Hes1, promoting proliferation and resistance (Shin et al., 2020). We extend this to HPH, confirming the MK-Notch2-Hes1 axis as a hypoxia-responsive module in PASMCs. Critically, lentiviral MK knockdown attenuated pathway activation, supporting MK’s position upstream of Notch2 activation. Functionally, hypoxia suppressed pro-apoptotic Bax and cleaved caspase-3 while elevating anti-apoptotic Bcl-2 (Figures 3B,G–I), reducing apoptosis (Figures 3C,F), and boosting proliferation (Figure 3E) and migration (Figures 3A,D). MK silencing restored the Bax/Bcl-2 ratio, elevated apoptosis, and curbed proliferation/migration, highlighting MK’s dual control over PASMC fate.

*In vivo*, AAV9-mediated MK knockdown in HPH rats validated these mechanisms. Lung tissues showed Notch2-Hes1 upregulation at protein (Figures 8A–D) and mRNA levels (Figures 8E–G), alongside mitochondrial apoptosis suppression (Figures 6B,G–I), mirroring *in vitro* findings. MK inhibition downregulated the pathway (∼60–80%), increased Bax/cleaved caspase-3, decreased Bcl-2, and boosted TUNEL-positive cells (Figures 6C,J), reducing vascular WT% (Figure 5G) and WA% (Figure 5H). Hemodynamics improved (mPAP ∼22 mmHg vs. ∼35 mmHg in HPH; Figure 5F), with alleviated inflammation, edema, and elastic lamina disruption (Figures 5A–E). Notably, this contrasts with MK’s protective role in ischemia-reperfusion injury, where deficiency worsens cardiomyocyte apoptosis (Majaj and Weckbach, 2022), underscoring MK’s context-specific functions: pro-survival in chronic hypoxia like HPH.

A novel aspect of our study is MK’s modulation of systemic oxidative stress (Figures 8H–K). HPH rats exhibited reduced antioxidant enzymes (SOD ∼30% lower, CAT ∼35% lower) and elevated damage markers (MDA ∼2-fold, LDH ∼1.5-fold), reflecting impaired redox balance. MK knockdown normalized these, suggesting MK exacerbates oxidative stress. Hypoxia-driven chemokines (e.g., CCR5) recruit inflammatory cells releasing IL-1β/IL-6/HMGB1 (Amsellem et al., 2014), while MK promotes macrophage/neutrophil infiltration and cytokine storms (Sanino et al., 2020). We propose a feed-forward loop in HPH: MK → inflammation → oxidative stress → PASMC proliferation/apoptosis resistance → remodeling. Disrupting MK breaks this cycle, offering multifaceted benefits beyond direct signaling. We acknowledge limitations in establishing MK’s precise relationship to Notch2-Hes1. Constitutively active NICD bypasses physiological receptor activation, so our rescue experiments support pathway compatibility rather than proving a strictly linear hierarchy. MK may also modulate parallel pathways. Future studies with Notch2-specific modulators or conditional knockouts are needed to clarify this architecture.

Current HPH therapies (phosphodiesterase-5 inhibitors, ERAs) modestly improve hemodynamics but fail to reverse remodeling or enhance survival (Boucly et al., 2025; Papadopoulos et al., 2025). By elucidating MK’s orchestration of Notch2-Hes1 and oxidative stress in HPH, our study identifies a high-yield target. Airway-delivered AAV9-shMK proved safe and effective, reducing PAP and remodeling without off-target effects.

Importantly, MK-targeted therapies could be integrated with existing pharmacological treatments to provide a more comprehensive approach to HPH management. While current drugs primarily induce vasodilation and symptom relief, MK inhibition directly targets the underlying vascular remodeling and apoptosis resistance that drive disease progression. Combining MK inhibitors—such as gene therapy vectors, monoclonal antibodies, or small molecules—with standard vasodilators may enhance therapeutic efficacy by simultaneously addressing hemodynamic abnormalities and pathological remodeling. This dual strategy holds promise for improving long-term outcomes and warrants investigation in future preclinical and clinical studies.

This supports developing MK inhibitors (e.g., antibodies, small molecules) as adjuncts or alternatives, potentially transforming HPH management.

Limitations include the lack of gain-of-function (e.g., MK overexpression) or Notch2-specific modulators to confirm causality. Oxidative stress regulation by MK—direct (e.g., enzyme expression) or indirect (via inflammation)—requires clarification via co-cultures or conditional knockouts. Future work with MK-Notch2 mouse models and agonists/antagonists will refine these mechanisms and advance clinical translation. Intratracheal AAV9 preferentially transduces alveolar epithelium, with limited PASMC and no GFP/α-SMA confirmation in this study. Vascular remodeling attenuation may thus reflect SMC-autonomous and/or epithelial paracrine effects. Future SMC-specific promoters (SM22α/MYH11) or conditional knockouts (e.g., Myh11-CreERT2; Mdkflox/flox) are needed to clarify the effector cells.

## 5 Conclusion

In summary, this study elucidates a hypoxia-driven pathogenic cascade in HPH: elevated MK activates the Notch2-Hes1 signaling axis to suppress mitochondrial apoptosis and promote PASMC proliferation, while also being associated with altered systemic antioxidant markers through inflammatory amplification. Additionally, through functional rescue experiments, this study further confirmed that MK is located upstream of the Notch2-Hes1 signaling axis. Forced activation of Notch2 could bypass MK deficiency to restore downstream Hes1 expression and cellular phenotypes, suggesting that MK acts upstream of Notch2-Hes1 signaling in this context rather than mere correlation, thereby providing a solid mechanistic foundation for targeting MK. Targeted MK inhibition via lentiviral shRNA *in vitro* and AAV9 delivery *in vivo* effectively disrupts this cascade, restoring apoptosis, curbing proliferation and migration, alleviating vascular remodeling, and normalizing redox balance—ultimately reducing mPAP. These findings illuminate MK’s central role in HPH pathogenesis and underscore its potential as a novel therapeutic target, paving the way for MK-specific interventions to improve clinical outcomes in this progressive disease.

## Data Availability

The original contributions presented in the study are included in the article/Supplementary Material, further inquiries can be directed to the corresponding authors.
