# Supplementary material for: Mechanistic investigation of Midkine-Notch2 signaling in suppressing pulmonary artery smooth muscle cell apoptosis and promoting vascular remodeling in hypoxic pulmonary hypertension
Source: Front Pharmacol. 2026 Jul 22;17:1788582. doi: 10.3389/fphar.2026.1788582 (PMC13437302; doi:10.3389/fphar.2026.1788582)
Supplement: Supplementary file 1 [file Supplementaryfile1.docx]

Supplementary Table 1 Design of Lentiviral shRNA

| Name |  |  | CDS | GC% | Seq ID No. |
| --- | --- | --- | --- | --- | --- |
| Midkine-shRNA(411) | NM_030859 | GAGCCGACTGCAAATACAAGT | 1..423 | 47.62% | 1 |
| Midkine-shRNA(412) | NM_030859 | CACCTCCAAGACCAAGTCAAA | 1..423 | 47.62% | 2 |
| Midkine-shRNA(413) | NM_030859 | CTGAAGAAGGCTCGGTACAAT | 1..423 | 47.62% | 3 |

Supplementary Table 2 Synthetic oligo information for lentivirus

| Name | 5’ | STEM | Loop | STEM | 3’ |
| --- | --- | --- | --- | --- | --- |
| Midkine-shRNA(411)-a | Ccgg | GAGCCGACTGCAAATACAAGT（Seq ID No.4） | CTCGAG | ACTTGTATTTGCAGTCGGCTC（Seq ID No.5） | TTTTTg |
| Midkine-shRNA(411)-b | Aattcaaaaa  （Seq ID No.6） | GAGCCGACTGCAAATACAAGT（Seq ID No.7） | CTCGAG | ACTTGTATTTGCAGTCGGCTC（Seq ID No.8） |  |
| Midkine-shRNA(412)-a | Ccgg | CACCTCCAAGACCAAGTCAAA（Seq ID No.9） | CTCGAG | TTTGACTTGGTCTTGGAGGTG（Seq ID No.10） | TTTTTg |
| Midkine-shRNA(412)-b | Aattcaaaaa  （Seq ID No.11） | CACCTCCAAGACCAAGTCAAA（Seq ID No.12） | CTCGAG | TTTGACTTGGTCTTGGAGGTG（Seq ID No.13） |  |
| Midkine-shRNA(413)-a | Ccgg | CTGAAGAAGGCTCGGTACAAT（Seq ID No.14） | CTCGAG | ATTGTACCGAGCCTTCTTCAG（Seq ID No.15） | TTTTTg |
| Midkine-shRNA(413)-b | Aattcaaaaa  （Seq ID No.16） | CTGAAGAAGGCTCGGTACAAT（Seq ID No.17） | CTCGAG | ATTGTACCGAGCCTTCTTCAG（Seq ID No.18） |  |

The carrier used in Table 2 is GV493. As shown in Figure 2, the sequence of the components is as follows: hU6-MCS-CBh-gcGFP-IRES-puromycin; the control insertion sequence is: TTCTCCGAACGTGTCACGT (Seq ID No. 19)

Supplementary Table 3 Design of AAV9 shRNA

| Name | GC% | Seq ID No. |
| --- | --- | --- |
| Midkine-shRNA(413)：CTGAAGAAGGCTCGGTACAAT | 47.62% | 20 |

Supplementary Table 4 AAV9 Synthesized oligo Information

| NO. | 5’ | STEM | Loop | STEM | 3’ |
| --- | --- | --- | --- | --- | --- |
| Midkine-shRNA(413)-a | ACCGG | CTGAAGAAGGCTCGGTACAAT（Seq ID No.21） | TTCAAGAGA | ATTGTACCGAGCCTTCTTCAG（Seq ID No.22） | TTTTT |
| Midkine-shRNA(413)-b | TCTAAAAAA | CTGAAGAAGGCTCGGTACAAT（Seq ID No.23） | TCTCTTGAA | ATTGTACCGAGCCTTCTTCAG（Seq ID No.24） | C |

In Table 4, the vector is GV478. As shown in Figure 17, the sequence of the elements: U6-MCS-CAG-EGFP. The control insertion sequence: CGCTGAGTACTTCGAAATGTC (Seq ID No. 25)

Supplementary Table 5 Primer Design for Target Protein and Housekeeping β-actin

| Nane | Sequence 5′ to 3′ |
| --- | --- |
| β-actin | F:GCCTTCCTTCCTGGGTATGGR |
|  | R:TCCGATTCAACTCATACTGC |
| BAX | F:CAGGATCGAGCAGAGAGGA |
|  | R:TGTCCAGTTCATCGCCAA |
| BCL-2 | F:AGCACATCCAATAAAAGCG |
|  | R:GGCCCGAAAGAGAGAAA |
| Caspase-3 | F:TGGCCCTGAAATACGAAG |
|  | R:ACTGCTCCTTTTGCTGTGA |
| Midkine | F:TTGCTACACCTAGTACCCAAAG |
|  | R:CTCGATAACAGGTATCAGGGTG |
| HES1 | F:GTAGGGGTCAGTGGCTTAGC |
|  | R:GAGGGTGGGGTAGGCTAAGA |
| Notch2 | F:TGCGAGACCAACATCAACGAGTG |
|  | R:TCAGGCAGAAGCAGAGGTAGGC |

Supplementary Table 6 Lentiviral Vector Information for Rat Notch2 NICD Overexpression

| Parameter | Details |
| --- | --- |
| Virus Name | Notch2-NICD-OE |
| Vector Backbone | GV492-Neo |
| Promoter | CMV |
| Insert | Rat Notch2 Intracellular Domain (NICD aa 1721-2431) |
| Protein Tag | Flag (for Western blot validation) |
| Fluorescent Marker | gcGFP (driven by CBh promoter) |
| Selection Resistance | IRES-Neomycin (confers G418 resistance) |
| Vector Element Order | CMV-NICD-Flag-CBh-gcGFP-IRES-Neomycin |
| Control Insert Sequence | TTCTCCGAACGTGTCACGT (Seq ID No. 19) |
| Supplier | Hainan Cool Pig Biotechnology Co. Ltd. |
| Catalog Number | CP-LV-rNotch2-NICD-Neo-023 |
| Virus Titer | 5×10⁷ TU/mL |

The Notch2-NICD-OE lentivirus utilizes the GV492-Neo vector backbone, with the CMV promoter driving expression of the rat Notch2 intracellular domain (NICD, amino acids 1721–2431). It carries a Flag tag for Western blot validation and contains the IRES-Neomycin gene conferring G418 resistance. The vector elements are arranged as follows: CMV-NICD-Flag-CBh-gcGFP-IRES-Neomycin. The control insertion sequence is TTCTCCGAACGTGTCACGT (Seq ID No. 19). The virus was purchased from Hainan Cool Pig Biotechnology Co., Ltd. (catalog number: CP-LV-rNotch2-NICD-Neo-023) at a titer of 5×10⁷ TU/mL.

Supplementary Table 7 Primer Sequences for NICD Overexpression Verification

| Primer Name | Direction | Sequence (5'→3') | Application |
| --- | --- | --- | --- |
| Flag-NICD-F | Forward | ATGGACTACAAAGACGATGACGACAAG | Flag-NICD fusion verification |
| Flag-NICD-R | Reverse | TTACTTGTCATCGTCATCCTTGTAGTC | Flag-NICD fusion verification |
| NICD-seq-F | Forward | TGCGAGACCAACATCAACGAGTG | NICD sequencing validation |
| NICD-seq-R | Reverse | TCAGGCAGAAGCAGAGGTAGGC | NICD sequencing validation |

Supplementary Table 8 Technical Parameters of Oxidative Stress Assay Kits

| **Assay** | **Catalog No.** | **Manufacturer** | **Detection Chemistry** | **Wavelength** | **Linear Range/Key Parameters** |
| --- | --- | --- | --- | --- | --- |
| **SOD** | A001-3 | Nanjing Jiancheng Bioengineering Institute | WST-1 method: xanthine oxidase-catalyzed superoxide anion generation oxidizes WST-1 to formazan; SOD activity quantified by inhibition rate | 450 nm | Standard curve: 2–50 U/mL; optimal inhibition rate 40–60%; 37°C incubation 20 min |
| **CAT** | A007-1-1 | Nanjing Jiancheng Bioengineering Institute | Ammonium molybdate method: CAT decomposes H₂O₂; residual H₂O₂ reacts with ammonium molybdate to form pale yellow complex | 405 nm | Slope reciprocal: 271 (spectrophotometer 0.5 cm path) or 235.65 (microplate reader 200 μL); strict 60-sec reaction at 37°C |
| **MDA** | A003-1 | Nanjing Jiancheng Bioengineering Institute | TBA colorimetric method: MDA condenses with thiobarbituric acid at 95°C for 40 min to form red product | 532 nm | Standard: 10 nmol/mL tetraethoxypropane; serum direct sampling 0.1 mL; tissue 10% homogenate |
| **LDH** | A020-2 | Nanjing Jiancheng Bioengineering Institute | Microplate method: LDH catalyzes lactate → pyruvate; pyruvate reacts with 2，4-dinitrophenylhydrazine to form red-brown hydrazone | 440 nm | Standard: 2 μmol/mL sodium pyruvate; recommended rat plasma dilution 50–100×; two-step 37°C incubation (15 min each) |

Supplementary Figure1. MK Inhibition Is Associated with Improved Antioxidant Profiles in Bronchoalveolar Lavage Fluid (BALF) from HPH Rats

**List of abbreviations**

analysis of variance (ANOVA)

damaged airway smooth muscle cells (ASMCs)

chronic obstructive pulmonary disease (COPD)

Cell Counting Kit-8 (CCK-8)

control (CONT)

catalase (CAT)

enzyme-linked immunosorbent assay (ELISA)

endothelin receptor antagonists (ERAs)

elastic Van Gieson (EVG)

green fluorescent protein (GFP)

Hypoxic pulmonary hypertension (HPH)

horseradish peroxide (HRP)

hematoxylin and eosin (H&E)

Immunofluorescence (IF)

lactate dehydrogenase (LDH)

Midkine (MK)

malondialdehyde (MDA)

multiplicities of infection (MOI)

mean PAP (mPAP)

Notch2 transmembrane form (NTM)

pulmonary arterial smooth muscle cell (PASMC)

pulmonary arterial smooth muscle cells (PASMCs)

pulmonary arterial pressure (PAP)

propidium iodide (PI)

phosphate-buffered saline (PBS)

quantitative real-time PCR (qPCR)

superoxide dismutase (SOD)

standard deviation (SD)

Terminal Deoxynucleotidyl Transferase dUTP Nick End Labeling (TUNEL)

Antibodies used in this study included: anti-MK (1:1000, rabbit polyclonal, Thermo Fisher, PA5150201), anti-Notch2 (1:1000, rabbit polyclonal, Thermo Fisher, PA5-22796), anti-Hes1 (1:1000, rabbit polyclonal, Servicebio, GB112254), anti-Bax (1:1000, rabbit polyclonal, Servicebio, GB114122), anti-Bcl-2 (1:1000, mouse monoclonal, Servicebio, GB154380), and anti‑caspase‑3 (1:1000, rabbit monoclonal, Servicebio, GB11532).

**Supplementary Method 1: Animal Model**

## Animal Model Establishment and Grouping

A cohort of thirty 6-week-old male Sprague-Dawley rats (160 ± 10 g) were housed under controlled conditions (20–24°C, 40–60% humidity, 12-h light/dark cycle) with ad libitum access to water and approximately 10 g of standard chow daily. Hypoxic pulmonary hypertension (HPH) was induced over 100 days by combining intratracheal instillation of lipopolysaccharide (LPS; 200 μg in 200 μL saline) on designated days with whole-body cigarette smoke (CS) exposure on non-instillation days. CS exposure was performed in a custom chamber using Hongmei brand cigarettes (13 mg tar/cigarette), with a daily regimen of smoke from 80 cigarettes divided into two 90-minute sessions. Successful HPH induction was confirmed by measuring mean pulmonary arterial pressure (mPAP ≥25 mmHg) via right jugular vein catheterization under sodium pentobarbital anesthesia (40 mg/kg, i.p.).

The HPH-positive rats (n=18) were randomly allocated into three groups (n=6 each): an HPH model group (intratracheal saline, 300 μL), an HPH + AAV9-Non-target group (non-targeting AAV9 vector, 1.5×10¹¹ vg/rat), and an HPH + AAV9-shRNA-MK group (MK-targeting AAV9, 1.5×10¹¹ vg/rat). All vectors were administered via a single intratracheal instillation on day 101. An age-matched control (CONT) group (n=6) of normoxic rats received equivalent saline. Four weeks post-treatment, all surgical procedures (hemodynamic measurement and tissue collection) were performed under deep anesthesia induced by sodium pentobarbital (40 mg/kg, i.p.), confirmed by loss of consciousness and absence of pedal reflex. Euthanasia was subsequently performed by cervical dislocation.

**Supplementary Method 2: Cell Culture**

The cells used in this experiment were derived from a rat pulmonary artery smooth muscle cell line (Purchased from Shanghai Qingqi Biotechnology Development Co., Ltd.). Cells were cultured in high-glucose Dulbecco’s Modified Eagle Medium (DMEM; Gibco) supplemented with 10% fetal bovine serum (FBS; Gibco), 100 U/mL penicillin, and 100 μg/mL streptomycin at 37°C in a 5% CO₂ humidified incubator. Cells were used between passages 3–6 and routinely passaged at 80–90% confluence and cryopreserved. To evaluate the knockdown efficiency, PASMCs were seeded in 6-well plates and transfected with three lentiviral shRNAs targeting MK (shRNA-411, shRNA-412, shRNA-413; each at 5×107 TU/mL) or a non-targeting scrambled shRNA control, using multiplicities of infection (MOI) of 50 in the presence of HitransG P/A enhancer (GeneChem, Shanghai, China). Transfection efficiency was qualitatively assessed 72 hours post-transduction by visualizing green fluorescent protein (GFP) expression via fluorescence microscopy. Stable polyclonal populations were then selected by treatment with 3 μg/mL puromycin until complete death of non-transfected control cells was observed, followed by maintenance in 0.5　μg/mL puromycin for 4 passages.

For functional assays, PASMCs were divided into four groups: CONT (normoxia, 20% O₂), Hypoxia (1% O₂, 5% CO₂, 37°C for 48 h using a hypoxia chamber [Thermo Fisher Scientific, Waltham, MA, USA]), Hypoxia + Non-target (hypoxia + scrambled shRNA lentivirus, MOI 50), and Hypoxia + shRNA-MK (hypoxia + MK-targeting shRNA lentivirus, MOI 50). To verify the MK→Notch2 hierarchy, MK shRNA-413-transduced PASMCs were co-infected with a constitutively active NICD lentivirus (neomycin-resistant, Hainan Coolpig Biotechnology Co., Ltd., Catalog No.: CP-LV-rNotch2-NICD-Neo-023). Dual-resistant cells were selected (puromycin 3 μg/mL, 72 h; G418 400 μg/mL, 96 h) and subjected to hypoxia (1% O₂, 48 h) for functional assays.Transfection efficiency and knockdown were confirmed by fluorescence microscopy and western blot at 48 h post-transfection.

**Supplementary Method 3:EVG Staining**

Paraffin sections (5 μm) were deparaffinized in two changes of environmentally friendly dewaxing solution (20 min each); rehydrated through anhydrous ethanol I (5 min), anhydrous ethanol II (5 min), and 75% ethanol (5 min); and then rinsed in running tap water. Sections were incubated in EVG working solution (prepared two days in advance by mixing EVG Stain A:B:C at a ratio of 5:2:2) for 5 min, rinsed in tap water, and differentiated using a 1:1 dilution of EVG Stain B under microscopic guidance until elastic fibers appeared purplish-black. Counterstaining was performed with fresh EVG solution (EVG Stain E [acid fuchsin]: EVG Stain D [picric acid] at a ratio of 9:1) for 1–3 min, followed by a water rinse, dehydration in anhydrous ethanol, clearing in xylene (two changes: 20 s and 5 min), and mounting with neutral balsam. Bright-field images were acquired using a light microscope to evaluate elastic fiber integrity (purplish-black) and collagen deposition (red) for pulmonary vascular remodeling.

**Supplementary Method 4: Terminal Deoxynucleotidyl Transferase dUTP Nick End Labeling (TUNEL) Assay**

Paraffin sections (5 μm ) were deparaffinized, rehydrated, treated with proteinase K (diluted 1:9 in PBS, 20 min at 37°C), and permeabilized with 0.5% Triton X-100 (20 min at room temperature). Apoptotic cells were labeled using an In Situ Cell Death Detection Kit (TUNEL, G1504, Servicebio, Wuhan, China) according to the manufacturer’s instructions (1 h at 37°C). Sections were counterstained with DAPI and imaged under fluorescence microscopy. The apoptotic index was quantified by randomly selecting three sections per group. For each section, five non-overlapping fields of view were captured at 200× magnification. The total number of nuclei (DAPI-positive, blue fluorescence) and TUNEL-positive nuclei (green fluorescence) within each field were counted. The apoptotic rate (%) was calculated as (TUNEL-positive nuclei / total DAPI-stained nuclei) × 100%.

Multiplex Fluorescence Staining

Paraffin‑embedded lung sections (5 μm) were dewaxed, rehydrated, and subjected to antigen retrieval in citrate buffer (pH 6.0; G1202, Servicebio) by microwave heating. After blocking with 10% goat serum (G1209, Servicebio) or 3% BSA (GC305006, Servicebio) for 30 min, sequential multiplex immunofluorescence staining was performed. Each staining cycle consisted of incubation with a primary antibody overnight at 4 °C, followed by an HRP‑conjugated secondary antibody (1:500; Jackson ImmunoResearch; 50 min) and tyramide signal amplification (PerkinElmer; 10 min). Antibodies were eluted between cycles using immunostaining elution buffer (G1266, Servicebio). The following primary antibodies were used: anti‑MK (1:100, rabbit polyclonal; PA5150201, Thermo Fisher), anti‑Notch2 (1:100, rabbit polyclonal; PA5‑22796, Thermo Fisher), anti‑Hes1 (1:1000, rabbit polyclonal; GB112254, Servicebio), anti‑Bax (1:400, rabbit polyclonal; GB114122, Servicebio), anti‑Bcl‑2 (1:200, mouse polyclonal; GB154380, Servicebio), and anti‑Caspase‑3 (1:700, rabbit monoclonal; GB11532, Servicebio). After all cycles, nuclei were counterstained with DAPI (G1012, Servicebio; 1:2000). Images were acquired using an upright fluorescence microscope (NIKON ECLIPSE C1) and analyzed for colocalization and intensity with ImageJ (5 fields/section, 3 sections/rat).
